# Malaria in African schoolchildren: options for control

**DOI:** 10.1016/j.trstmh.2008.01.010

**Published:** 2008-04

**Authors:** Simon Brooker, Siân Clarke, Robert W. Snow, Donald A.P. Bundy

**Affiliations:** a Department of Infectious and Tropical Diseases, London School of Hygiene and Tropical Medicine, London, UK; b Malaria Public Health and Epidemiology Group, Centre for Geographic Medicine, KEMRI/Wellcome Trust Research Laboratories, Nairobi, Kenya; c Centre for Tropical Medicine, University of Oxford, Oxford, UK; d Human Development Division, The World Bank, Washington, DC, USA

**Keywords:** Malaria, Schools, Schoolchildren, Long-lasting insecticidal nets, Chemoprevention, Africa

## Abstract

Intensified malaria control efforts among young African children may increase disease risks among older children who attend school and whose education may be impaired by malaria. However, there is currently no consensus as to the approach to malaria control in schools, with relevant intervention strategies varying according to patterns of malaria transmission. Life skills messages regarding prevention and accessing prompt treatment are important everywhere. Providing free bed nets to schoolchildren may bring individual and community benefits and should be widely promoted. New approaches to school-based chemoprevention and treatment may also be able to play an important role in school-based malaria control, although these require further investigation.

Intensified malaria control efforts among young African children may result in later acquisition of exposure-dependent immunity and potentially increases the risk for children of school age and above. This epidemiological transition is occurring at a time when more children than ever before are attending school and when governments are increasingly recognising the importance of child health for educational achievement (Bundy et al., 2000). Surprisingly little is known about the burden of malaria in African schoolchildren, but the available evidence suggests that malaria causes up to 50% of all deaths in this age group, is an important major contributor to anaemia and may have profound consequences for learning and educational achievement (Lalloo et al., 2006).

To help enhance education opportunities, many school health programmes in Africa already provide schoolchildren with health education and health services, including regular delivery of anthelminthics and micronutrients. This existing infrastructure could deliver school-based malaria interventions, but there is currently no consensus as to the optimal approach. Here we consider three possible options as well as what is known about their implementation in practice.

First, long-lasting insecticidal nets (LLIN) are currently promoted among high-risk groups, especially young children and pregnant women. Recent evidence shows that protecting all community members yields enhanced benefits in terms of health and social equity (Killeen et al., 2007), and the WHO now recommends that LLINs should be used by everyone, including schoolchildren. For this to happen there would have to be a dramatic increase in coverage from the current very low levels of use in schoolchildren. This implies providing free LLINs to schoolchildren, encouraging the very large number of residential (boarding) schools to provide nets in dormitories, and supporting the strategy with skills-based health education, which can help to ensure that children develop the knowledge, attitudes and skills necessary to reduce their risk from malaria.

Health education to promote appropriate behaviour is a traditional and natural activity by which schools contribute to disease prevention, but new approaches to chemoprevention may also be able to play an important role. Historically, school-based delivery of malaria chemoprophylaxis was associated with significant reductions in malaria-related morbidity and mortality as well as improvements in educational outcomes, but it fell out of use in Africa with the development of malaria drug resistance. More recent evidence elsewhere suggests that weekly chemoprophylaxis can improve school examination scores (for a review see Lalloo et al., 2006) but tends to be compromised by declining compliance and coverage over time. An alternative strategy, already proven effective for protecting the health of women in pregnancy, is intermittent preventive treatment (IPT). In Kenya, mass administration of a full therapeutic course of an antimalarial drug to schoolchildren once a term, irrespective of infection status, dramatically reduced malaria parasitaemia, almost halved the rates of anaemia and significantly improved cognitive ability (Clarke et al., 2006).

The third promising intervention option is presumptive treatment of schoolchildren by teachers in schools. Whilst this approach might raise questions about the reliability of diagnosis by non-health personnel and the long-term motivation of teachers to play a health role, a large-scale programmatic evaluation in Malawian schools showed that treatment was associated with a reduction in malaria-specific mortality in schoolchildren (Pasha et al., 2003).

With all these interventions there is a special role for schools in addressing the needs of girls. Malaria in pregnancy is already recognised as an important risk factor, and schools have particular responsibility to girls as they enter the reproductive age range. At a minimum there is a need for all these options to avoid the accidental treatment of pregnant schoolgirls, but more generally the schools have an important role, through the health education curriculum, of teaching girls about the benefits of reducing early and unplanned pregnancy, of attending antenatal health services and of accessing LLINs and IPT.

The same suite of school-based strategies will not be relevant everywhere. Life skills messages about the use of LLINs, the early recognition of malaria and how to access prompt treatment should probably be part of health education in all transmission settings, whereas IPT may be relevant only in high transmission areas. By contrast, in epidemic-prone settings neither LLINs nor IPT would be appropriate; instead schools may provide useful sentinels for epidemic detection and strengthening of drug supplies at health facilities. The selection of cost-effective intervention options will need to be guided by an informed understanding of the epidemiology and geography of malaria, and choices may alter with changes in the patterns of malaria transmission and clinical immunity.

## Funding

Additional funding was provided by the Norwegian Education Trust Fund and the multi-donor Education Program Development Fund, both administered by the Africa Region Human Development Department of the World Bank. The results reported here contributed to the World Bank Africa Program for Education Impact Evaluation and the Malaria Impact Evaluation Program (http://go.worldbank.org/E70Y4QHZW0) both of which seek to strengthen a result-based approach to national programs within the framework of the Education for All Fast Track Initiative to advance the education MDGs. SB is supported by a Wellcome Trust Research Career Development Fellowship (081673) and RWS by a Wellcome Trust Principal Research Fellowship (079080).

## Conflicts of interest

None declared.

## Ethical approval

Not required.
